# Learning Pomset Automata

**DOI:** 10.1007/978-3-030-71995-1_26

**Published:** 2021-03-23

**Authors:** Gerco van Heerdt, Tobias Kappé, Jurriaan Rot, Alexandra Silva

**Affiliations:** 8 University of Oxford, Oxford, UK; 9 Sorbonne Université - LIP6, Paris, France; 10 University College London, London, UK; 11 Cornell University, Ithaca, NY USA; 12 Radboud University, Nijmegen, The Netherlands

## Abstract

We extend the $\mathtt {L}^{\!\star }$ algorithm to learn bimonoids recognising pomset languages. We then identify a class of pomset automata that accepts precisely the class of pomset languages recognised by bimonoids and show how to convert between bimonoids and automata.

## 1 Introduction

Automata learning algorithms are useful in automated inference of models, which is needed for verification of hardware and software systems. In *active* learning, the algorithm interacts with a system through tests and observations to produce a model of the system's behaviour. One of the first active learning algorithms proposed was $\mathtt{L}^\star$, due to Dana Angluin [2], which infers a minimal deterministic automaton for a target regular language. $\mathtt{L}^\star$ has been used in a range of verification tasks, including learning error traces in a program [5]. For more advanced verification tasks, richer automata types are needed and $\mathtt{L}^\star$ has been extended to e.g. input-output [1], register [20], and weighted automata [16]. None of the existing extensions can be used in analysis of concurrent programs.

Partially ordered multisets (pomsets) [13,12] are basic structures used in the modeling and semantics of concurrent programs. Pomsets generalise words, allowing to capture both the sequential and the parallel structure of a trace in a concurrent program. Automata accepting pomset languages are therefore useful to study the operational semantics of concurrent programs—see, for instance, work on concurrent Kleene algebra [17,26,21,24].

In this paper, we propose an active learning algorithm for a class of pomset automata. The approach is algebraic: we consider languages of pomsets recognised by bimonoids [28] (which we shall refer to as pomset recognisers). This can be thought of as a generalisation of the classical approach to language theory of using monoids as word acceptors: bimonoids have an extra operation that models parallel composition in addition to sequential. The two operations give rise to a complex branching structure that makes the learning process non-trivial.

* This work was partially supported by the ERC Starting Grant ProFoundNet (679127) and the EPSRC Standard Grant CLeVer (EP/S028641/1). The authors thank Matteo Sammartino for useful discussions.

The key observation is that pomset recognisers are tree automata whose algebraic structure satisfies additional equations. We extend tree automata learning algorithms [7,8,31] to pomset recognisers. The main challenge is to ensure that intermediate hypotheses in the algorithm are valid pomset recognisers, which is essential in practical scenarios where the learning process might not run to the very end, returning an approximation of the system under learning. This requires equations of bimonoids to be correctly propagated and preserved in the core data structure of the algorithm—the observation table. The proof of termination, in analogy to $\mathtt{L}^\star$, relies on the existence of a canonical pomset recogniser of a language, which is based on its syntactic bimonoid. The steps of the algorithm provide hypotheses that get closer in size to the canonical recogniser.

Finally, we bridge the learning algorithm to pomset automata [21,22] by providing two constructions that enable us to seamlessly move between pomset recognisers and pomset automata. Note that although bimonoids provide a useful formalism to denote pomset languages, which is amenable to the design of the learning algorithm, they enforce a redundancy that is not present in pomset automata: whereas a pomset automaton processes a pomset from left to right in sequence, one letter per branch at a time, a bimonoid needs to be able to take the pomset represented as a binary tree in any way and process it bottom-up. This requirement of different decompositions leading to the same result makes bimonoids in general much larger than pomset automata and hence the latter are, in general, a more efficient representation of a pomset language.

The rest of the paper is organised as follows. We conclude this introductory section with a review of relevant related work. Section 2 contains the basic definitions on pomsets and pomset recognisers. The learning algorithm for pomset recognisers appears in Section 3, including proofs to ensure termination and invariant preservation. Section 4 presents constructions to translate between (a class of) pomset automata and pomset recognisers. We conclude with discussion of further work in Section 5. Omitted proofs appear in the extended version [15].

*Related Work.* There is a rich literature on adaptations and extensions of $\mathtt{L}^\star$ from deterministic automata to various kinds of models, see, e.g., [34,18] for an overview. To the best of our knowledge, this paper is the first to provide an active learning algorithm for pomset languages recognised by finite bimonoids.

Our algorithm learns an algebraic recogniser. Urbat and Schröder [33] provide a very general learning approach for languages recognised by algebras for monads [4,32], based on a reduction to categorical automata, for which they present an $\mathtt{L}^\star$-type algorithm. Their reduction gives rise to an infinite alphabet in general, so tailored work is needed for deriving algorithms and finite representations. This can be done for instance for monoids, recognising regular languages, but it is not clear how this could extend to pomset recognisers. We present a direct learning algorithm for bimonoids, which does not rely on any encoding.

Our concrete learning algorithm for bimonoids is closely related to learning approaches for bottom-up tree automata [7,8,31]: pomset languages can be viewed as tree languages satisfying certain equations. Incorporating these equations turned out to be a non-trivial task, which requires additional checks on the observation table during execution of the algorithm.

Conversion between recognisers and automata for a pomset language was first explored by Lodaya and Weil [28,27]. Their results relate the expressive power of these formalisms to *sr-expressions*. As a result, converting between recognisers and automata using their construction uses an sr-expression as an intermediate representation, increasing the resulting state space. Our construction, however, converts recognisers directly to pomset automata, which keeps the state space relatively small. Moreover, Lodaya and Weil work focus on pomset languages of *bounded width*, i.e., with an upper bound on the number of parallel events. In contrast, our conversions work for all recognisable pomset languages (and a suitable class of pomset automata), including those of unbounded width.

Ésik and Németh [9] considered automata and recognisers for *biposets*, i.e., sp-pomsets without commutativity of parallel composition. They equate languages recognised by *bisemigroups* (bimonoids without commutativity or units) with those accepted by *parenthesizing automata*. Our equivalence is similar in structure, but relates a subclass of pomset automata to bimonoids instead. The results in this paper can easily be adapted to learn representations of biposet languages using bisemigroups, and convert those to parenthesizing automata.

## 2 Pomset Recognisers

Throughout this paper we fix a finite *alphabet* $\Sigma$ and assume $\Box \notin \Sigma$. When defining sets parameterised by a set $X$, say $\mathsf{S}(X)$, we may use $\mathsf{S}$ to refer to $\mathsf{S}(\Sigma)$.

We recall pomsets [12,13], a generalisation of words that model concurrent traces. A *labelled poset* over $X$ is a tuple $\mathbf{u} = \langle S_\mathbf{u}, \leq_\mathbf{u}, \lambda_\mathbf{u} \rangle$, where $S_\mathbf{u}$ is a finite set (the *carrier* of $\mathbf{u}$), $\leq_\mathbf{u}$ is a partial order on $S_\mathbf{u}$ (the *order* of $\mathbf{u}$), and $\lambda_\mathbf{u} \colon S_\mathbf{u} \to X$ is a function (the *labelling* of $\mathbf{u}$). Pomsets are labelled posets up to isomorphism.

**Definition 1 (Pomsets).** *Let* $\mathbf{u}, \mathbf{v}$ *be labelled posets over* $X$*. An* embedding *of* $\mathbf{u}$ *in* $\mathbf{v}$ *is an injection* $h : S_\mathbf{u} \to S_\mathbf{v}$ *such that* $\lambda_\mathbf{v} \circ h = \lambda_\mathbf{u}$ *and* $s \leq_\mathbf{u} s'$ *if and only if* $h(s) \leq_\mathbf{v} h(s')$*. An* isomorphism *is a bijective embedding whose inverse is also an embedding. We say* $\mathbf{u}$ *is* isomorphic *to* $\mathbf{v}$*, denoted* $\mathbf{u} \cong \mathbf{v}$*, if there exists an isomorphism between* $\mathbf{u}$ *and* $\mathbf{v}$*. A* pomset *over* $X$ *is an isomorphism class of labelled posets over* $X$*, i.e.,* $[\mathbf{v}] = \{\mathbf{u} : \mathbf{u} \cong \mathbf{v}\}$*. When* $u = [\mathbf{u}]$ *and* $v = [\mathbf{v}]$ *are pomsets,* $u$ *is a* subpomset *of* $v$ *when there exists an embedding of* $\mathbf{u}$ *in* $\mathbf{v}$*.*

When two pomsets are in scope, we tacitly assume that they are represented by labelled posets with disjoint carriers. We write 1 for the empty pomset. When $\mathsf{a} \in X$, we write $\mathsf{a}$ for the pomset represented by the labelled poset whose sole element is labelled by $\mathsf{a}$. Pomsets can be composed in sequence and in parallel:

**Definition 2 (Pomset composition).** *Let* $u = [\mathbf{u}]$ *and* $v = [\mathbf{v}]$ *be pomsets over* $X$*. We write* $u \parallel v$ *for the* parallel composition *of* $u$ *and* $v$*, which is the pomset over* $X$ *represented by the labelled poset*

$$\mathbf{u} \parallel \mathbf{v} = \langle S_\mathbf{u} \cup S_\mathbf{v},\ \leq_\mathbf{u} \cup \leq_\mathbf{v},\ \lambda_\mathbf{u} \cup \lambda_\mathbf{v} \rangle$$

*Similarly, we write* $u \cdot v$ *for the* sequential composition *of* $u$ *and* $v$*, that is, the pomset represented by the labelled poset*

$$\mathbf{u} \cdot \mathbf{v} = \langle S_{\mathbf{u}} \cup S_{\mathbf{v}},\ \ \leq_{\mathbf{u}} \cup \leq_{\mathbf{v}} \cup S_{\mathbf{u}} \times S_{\mathbf{v}},\ \ \lambda_{\mathbf{u}} \cup \lambda_{\mathbf{v}} \rangle$$

*We may elide the dot for sequential composition, for instance writing* $\mathtt{ab}$ *for* $\mathtt{a} \cdot \mathtt{b}$.

The pomsets we use can be built using sequential and parallel composition.

**Definition 3 (Series-parallel pomsets).** *The set of* series-parallel pomsets (sp-pomsets) *over* $X$*, denoted* $\mathsf{SP}(X)$*, is the smallest set such that* $1 \in \mathsf{SP}(X)$ *and* $\mathtt{a} \in \mathsf{SP}(X)$ *for every* $\mathtt{a} \in X$*, closed under parallel and sequential composition.*

Concurrent systems admit executions of operations that are not only ordered in sequence but also allow parallel branches. An algebraic structure consisting of both a sequential and a parallel composition operation, with a shared unit, is called a *bimonoid*. Formally, its definition is as follows.

**Definition 4 (Bimonoid).** *A* bimonoid *is a tuple* $\langle M, \odot, \varobar, \mathbf{1} \rangle$ *where*

- $M$ *is a set called the* carrier *of the bimonoid,*
- $\odot$ *is a binary associative operation on* $M$*,*
- $\varobar$ *is a binary associative and commutative operation on* $M$*, and*
- $\mathbf{1} \in M$ *is a unit for both* $\odot$ *(on both sides) and* $\varobar$*.*

*Bimonoid homomorphisms are defined in the usual way.*

Given a set $X$, the *free bimonoid* [12] over $X$ is $\langle \mathsf{SP}(X), \cdot, \parallel, 1 \rangle$. The fact that it is free means that for every function $f \colon X \to M$ for a given bimonoid $\langle M, \odot, \varobar, \mathbf{1}_M \rangle$ there exists a unique bimonoid homomorphism $f^\sharp \colon \mathsf{SP}(X) \to M$ such that the restriction of $f^\sharp$ to $X$ is $f$.

Just as monoids can recognise words, bimonoids can recognise pomsets [28]. A bimonoid together with the witnesses of recognition is a *pomset recogniser*.

**Definition 5 (Pomset recogniser).** *A* pomset recogniser *is a tuple* $\mathcal{R} = \langle M, \odot, \varobar, \mathbf{1}, i, F \rangle$ *where* $\langle M, \odot, \varobar, \mathbf{1} \rangle$ *is a bimonoid,* $i \colon \Sigma \to M$*, and* $F \subseteq M$*. The* language recognised *by* $\mathcal{R}$ *is given by* $\mathcal{L}_{\mathcal{R}} = \{u \in \mathsf{SP} : i^\sharp(u) \in F\} \subseteq \mathsf{SP}$.

*Example 6.* Suppose a program consists of a loop, where each iteration runs actions $\mathtt{a}$ and $\mathtt{b}$ in parallel. We can describe the behaviour of this program by

$$\mathcal{L} = \{\mathtt{a} \parallel \mathtt{b}\}^* = \{1, \mathtt{a} \parallel \mathtt{b}, (\mathtt{a} \parallel \mathtt{b}) \cdot (\mathtt{a} \parallel \mathtt{b}), \ldots\}$$

We can describe this language using a pomset recogniser, as follows. Let $M = \{q_{\mathtt{a}}, q_{\mathtt{b}}, q_1, q_\bot, \mathbf{1}\}$, and let $\odot$ and $\varobar$ be the operations on $M$ given by

$$q \odot q' = \begin{cases} q & q' = \mathbf{1} \\ q' & q = \mathbf{1} \\ q_1 & q = q' = q_1 \\ q_\bot & \text{otherwise} \end{cases} \qquad\qquad q \varobar q' = \begin{cases} q & q' = \mathbf{1} \\ q' & q = \mathbf{1} \\ q_1 & \{q, q'\} = \{q_{\mathtt{a}}, q_{\mathtt{b}}\} \\ q_\bot & \text{otherwise} \end{cases}$$

A straightforward proof verifies that $\langle M, \odot, \oplus, \mathbf{1} \rangle$ is a bimonoid.

We set $i(\mathtt{a}) = q_\mathtt{a}$, $i(\mathtt{b}) = q_\mathtt{b}$, and $F = \{\mathbf{1}, q_1\}$. Now, for $n > 0$:

$$i^\sharp(\underbrace{(\mathtt{a} \parallel \mathtt{b}) \cdots (\mathtt{a} \parallel \mathtt{b})}_{n \text{ times}}) = \underbrace{(i(\mathtt{a}) \parallel i(\mathtt{b})) \odot \cdots \odot (i(\mathtt{a}) \parallel i(\mathtt{b}))}_{n \text{ times}} = \underbrace{q_1 \odot \cdots \odot q_1}_{n \text{ times}} = q_1$$

No other pomsets are mapped to $q_1$; hence, $\langle M, \odot, \oplus, \mathbf{1}, i, F \rangle$ accepts $\mathcal{L}$.

*Example 7.* Suppose a program solves a problem recursively, such that the recursive calls are performed in parallel. In that case, the program would either perform the base action $\mathtt{b}$, or some preprocessing action $\mathtt{a}$ followed by running two copies of itself in parallel. This behaviour can be described by the smallest pomset language $\mathcal{L}$ satisfying the following inference rules:

$$\frac{}{\mathtt{b} \in \mathcal{L}} \qquad\qquad \frac{u, v \in \mathcal{L}}{\mathtt{a} \cdot (u \parallel v) \in \mathcal{L}}$$

This language can be described by a pomset recogniser. Let our carrier set be $M = \{q_\mathtt{a}, q_\mathtt{b}, q_1, q_\bot, \mathbf{1}\}$, and let $\odot$ and $\oplus$ be the operations on $M$ given by

$$q \odot q' = \begin{cases} q & q' = \mathbf{1} \\ q' & q = \mathbf{1} \\ q_\mathtt{b} & q = q_\mathtt{a}, q' = q_1 \\ q_\bot & \text{otherwise} \end{cases} \qquad\qquad q \oplus q' = \begin{cases} q & q' = \mathbf{1} \\ q' & q = \mathbf{1} \\ q_1 & q = q' = q_\mathtt{b} \\ q_\bot & \text{otherwise} \end{cases}$$

$\langle M, \odot, \oplus, \mathbf{1} \rangle$ is a bimonoid, $F = \{q_\mathtt{b}\}$, and $i \colon \Sigma \to M$ is given by setting $i(\mathtt{a}) = q_\mathtt{a}$ and $i(\mathtt{b}) = q_\mathtt{b}$. One can then show that $\langle M, \odot, \oplus, \mathbf{1}, i, F \rangle$ accepts $\mathcal{L}$.

*Pomset contexts* are used to describe the behaviour of individual elements in a pomset recogniser. Formally, the set of pomset contexts over a set $X$ is given by $\mathsf{PC}(X) = \mathsf{SP}(X \cup \{\square\})$. Here the element $\square$ acts as a placeholder, where a pomset can be plugged in: given a context $c \in \mathsf{PC}(X)$ and $t \in \mathsf{SP}(X)$, let $c[t] \in \mathsf{SP}(X)$ be obtained by substituting $t$ for $\square$ in $c$.

## 3 Learning Pomset Recognisers

In this section we present our algorithm to learn pomset recognisers from an oracle (*the teacher*) that answers *membership* and *equivalence queries*. A membership query consists of a pomset, to which the teacher replies whether that pomset is in the language; an equivalence query consists of a *hypothesis* pomset recogniser, to which the teacher replies *yes* if it is correct or *no* with a counterexample—a pomset incorrectly classified by the hypothesis—if it is not.

A pomset recogniser is essentially a tree automaton, with the additional constraint that its algebraic structure satisfies the bimonoid axioms. Our algorithm is therefore relatively close to tree automata learning—in particular Drewes and Högberg [7,8]—but there are several key differences: we optimise the algorithm by taking advantage of the bimonoid axioms, and at the same time need to ensure that the hypotheses generated by the learning process satisfy those axioms.

### 3.1 Observation Table

We fix a target language $\mathcal{L} \subseteq \mathsf{SP}$ throughout this section. As in the original $\mathtt{L}^\star$ algorithm, the state of the learner throughout a run of the algorithm is given by a data structure called the *observation table*, which collects information about $\mathcal{L}$. The table contains rows indexed by pomsets, representing the state reached by the correct pomset recogniser after reading that pomset; and columns indexed by pomset contexts, used to approximately indentify the behaviour of each state. To represent the additional rows needed to approximate the pomset recogniser structure, we use the following definition. Given $U \subseteq \mathsf{SP}$, we define

$$U^+ = \Sigma \cup \{u \cdot v : u, v \in U\} \cup \{u \parallel v : u, v \in U\} \subseteq \mathsf{SP}.$$

**Definition 8 (Observation table).** *An* observation table *is a pair $\langle S, E\rangle$, with $S \subseteq \mathsf{SP}$ subpomset-closed and $E \subseteq \mathsf{PC}$ such that $1 \in S$ and $\square \in E$. These sets induce the function $\mathsf{row}_{\langle S,E\rangle} : S \cup S^+ \to 2^E$: $\mathsf{row}_{\langle S,E\rangle}(s)(e) = 1 \iff e[s] \in \mathcal{L}$. We often write* $\mathsf{row}$ *instead of* $\mathsf{row}_{\langle S,E\rangle}$ *when $S$ and $E$ are clear from the context.*

We depict observation tables, or more precisely $\mathsf{row}$, as two separate tables with rows in $S$ and $S^+ \setminus S$ respectively, see for instance Example 9 below.

The goal of the learner is to extract a *hypothesis* pomset recogniser from the rows in the table. More specifically, the carrier of the underlying bimonoid of the hypothesis will be given by the rows indexed by pomsets in $S$. The structure on the rows is obtained by transferring the structure of the row labels onto the rows (e.g., $\mathsf{row}(s) \odot \mathsf{row}(t) = \mathsf{row}(s \cdot t)$), but this is not well-defined unless the table satisfies *closedness*, *consistency*, and *associativity*. Closedness and consistency are standard in $\mathtt{L}^\star$, whereas associativity is a new property specific to bimonoid learning. We discuss each of these properties next, also including *compatibility*, a property that is used to show minimality of hypotheses.

The first potential issue is a closedness defect: this is the case when a composed row, indexed by an element of $S^+$, is not indexed by a pomset in $S$.

*Example 9 (Table not closed).* Recall $\mathcal{L} = \{\mathtt{a} \parallel \mathtt{b}\}^*$ from Example 6, and suppose $S = \{1, \mathtt{a}, \mathtt{b}\}$ and $E = \{\square, \mathtt{a} \parallel \square, \square \parallel \mathtt{b}\}$. The induced table is

| $S$ | $\square$ | $\mathtt{a} \parallel \square$ | $\square \parallel \mathtt{b}$ |
|---|---|---|---|
| 1 | 1 | 0 | 0 |
| a | 0 | 0 | 1 |
| b | 0 | 1 | 0 |

| $S^+ \setminus S$ | $\square$ | $\mathtt{a} \parallel \square$ | $\square \parallel \mathtt{b}$ |
|---|---|---|---|
| aa | 0 | 0 | 0 |
| ab | 0 | 0 | 0 |
| ba | 0 | 0 | 0 |
| bb | 0 | 0 | 0 |
| a ∥ a | 0 | 0 | 0 |
| a ∥ b | 1 | 0 | 0 |
| b ∥ b | 0 | 0 | 0 |

The carrier of the hypothesis bimonoid is $M = \{\mathsf{row}(1), \mathsf{row}(\mathtt{a}), \mathsf{row}(\mathtt{b})\}$, but the composition $\mathsf{row}(\mathtt{a}) \odot \mathsf{row}(\mathtt{a})$ cannot be defined since $\mathsf{row}(\mathtt{aa}) \notin M$.

The absence of the issue described above is captured with *closedness*.

**Definition 10 (Closed table).** *An observation table* $\langle S, E\rangle$ *is* closed *if for all* $t \in S^+$ *there exists* $s \in S$ *such that* $\mathsf{row}(s) = \mathsf{row}(t)$.

Another issue that may occur is that the same row being represented by different index pomsets leads to an inconsistent definition of the structure. The absence of this issue is referred to as *consistency.*

**Definition 11 (Consistent table).** *An observation table* $\langle S, E\rangle$ *is* consistent *if for all* $s_1, s_2 \in S$ *such that* $\mathsf{row}(s_1) = \mathsf{row}(s_2)$ *we have for all* $t \in S$ *that*

$$\mathsf{row}(s_1 \cdot t) = \mathsf{row}(s_2 \cdot t) \qquad \mathsf{row}(t \cdot s_1) = \mathsf{row}(t \cdot s_2) \qquad \mathsf{row}(s_1 \parallel t) = \mathsf{row}(s_2 \parallel t).$$

Whenever closedness and consistency hold, one can define sequential and parallel composition operations on the rows of the table. However, these operations are not guaranteed to be associative, as we show with the following example.

*Example 12 (Table not associative).* Consider $\mathcal{L} = \{\mathtt{a}u : u \in \{\mathtt{b}\}^*\}$ over $\Sigma = \{\mathtt{a}, \mathtt{b}\}$, and suppose $S = \{1, \mathtt{a}, \mathtt{b}\}$ and $E = \{\square, \square\mathtt{a}\}$. The induced table is:

| | $\square$ | $\square$a |
|---|---|---|
| 1 | 0 | 1 |
| a | 1 | 0 |
| b | 0 | 0 |

| | $\square$ | $\square$a |
|---|---|---|
| aa | 0 | 0 |
| ab | 1 | 0 |
| ba | 0 | 0 |
| bb | 0 | 0 |
| a ‖ a | 0 | 0 |
| a ‖ b | 0 | 0 |
| b ‖ b | 0 | 0 |

This table does not lead to an associative sequential operation on rows:

$$(\mathsf{row}(\mathtt{a}) \odot \mathsf{row}(\mathtt{b})) \odot \mathsf{row}(\mathtt{a}) = \mathsf{row}(\mathtt{ab}) \odot \mathsf{row}(\mathtt{a}) = \mathsf{row}(\mathtt{a}) \odot \mathsf{row}(\mathtt{a}) = \mathsf{row}(\mathtt{aa})$$
$$\neq \mathsf{row}(\mathtt{ab}) = \mathsf{row}(\mathtt{a}) \odot \mathsf{row}(\mathtt{b}) = \mathsf{row}(\mathtt{a}) \odot \mathsf{row}(\mathtt{ba}) = \mathsf{row}(\mathtt{a}) \odot (\mathsf{row}(\mathtt{b}) \odot \mathsf{row}(\mathtt{a})).$$

To prevent this issue we enforce the following additional property:

**Definition 13 (Associative table).** *Let* $\heartsuit \in \{\cdot, \parallel\}$. *An observation table* $\langle S, E\rangle$ *is* $\heartsuit$-associative *if for all* $s_1, s_2, s_3, s_l, s_r \in S$ *with* $\mathsf{row}(s_l) = \mathsf{row}(s_1 \heartsuit s_2)$ *and* $\mathsf{row}(s_r) = \mathsf{row}(s_2 \heartsuit s_3)$ *we have* $\mathsf{row}(s_l \heartsuit s_3) = \mathsf{row}(s_1 \heartsuit s_r)$. *An observation table is* associative *if it is both* $\cdot$*-associative and* $\parallel$*-associative.*

The table from Example 12 is *not* $\cdot$-associative: we have $\mathsf{row}(\mathtt{a}) = \mathsf{row}(\mathtt{ab})$ and $\mathsf{row}(\mathtt{b}) = \mathsf{row}(\mathtt{ba})$ but $\mathsf{row}(\mathtt{aa}) \neq \mathsf{row}(\mathtt{ab})$.

Putting the above definitions of closedness, consistency and associativity of tables together, we have the following result for constructing a hypothesis.

**Lemma 14 (Hypothesis).** *A closed, consistent and associative table* $\langle S, E\rangle$ *induces a* hypothesis *pomset recogniser* $\mathcal{H} = \langle H, \odot_H, \oplus_H, \mathbf{1}_H, i_H, F_H\rangle$ *where*

$$H = \{\mathsf{row}(s) : s \in S\} \qquad \mathsf{row}(s_1) \odot_H \mathsf{row}(s_2) = \mathsf{row}(s_1 \cdot s_2)$$

$$\mathsf{row}(s_1) \oplus_H \mathsf{row}(s_2) = \mathsf{row}(s_1 \parallel s_2) \qquad \mathbf{1}_H = \mathsf{row}(1) \qquad i_H(\mathtt{a}) = \mathsf{row}(\mathtt{a})$$

$$F_H = \{\mathsf{row}(s) : s \in S, \mathsf{row}(s)(\square) = 1\}.$$

*Proof.* The operations $\odot_H$ and $\oplus_H$ are well-defined by closedness and consistency, and $\mathbf{1}_H$ is well-defined because $1 \in S$ by the observation table definition. Commutativity of $\oplus_H$ follows from commutativity of $\parallel$, and similarly that $\mathbf{1}_H$ is a unit for both operations follows from 1 being a unit. Associativity follows by associativity of the table (it does *not* follow from $\cdot$ and $\parallel$ being associative: given elements $s_1, s_2, s_3 \in S$, $s_1 \cdot s_2 \cdot s_3$ is not necessarily present in $S \cup S^+$). □

Since a hypothesis is constructed from an observation table $\langle S, E \rangle$ that records for given $s \in S$ and $e \in E$ whether $e[s]$ is accepted by the language or not, one would expect that the hypothesis classifies those pomsets

$$T_{\langle S,E \rangle} = \{e[s] : s \in S, e \in E\}$$

correctly. This is not necessarily the case, as we show in the following example.

*Example 15.* Consider the language $\mathcal{L}$ from Example 7, and let $S = \{1, \mathtt{b}\}$ and $E = \{\square, \mathtt{a}(\square \parallel \mathtt{b})\}$. The induced table is

| | $\square$ | $\mathtt{a}(\square \parallel \mathtt{b})$ |
|---|---|---|
| 1 | 0 | 0 |
| $\mathtt{b}$ | 1 | 1 |

| | $\square$ | $\mathtt{a}(\square \parallel \mathtt{b})$ |
|---|---|---|
| $\mathtt{a}$ | 0 | 0 |
| $\mathtt{bb}$ | 0 | 0 |
| $\mathtt{b} \parallel \mathtt{b}$ | 0 | 0 |

From this closed, consistent, and associative table we obtain a hypothesis pomset recogniser that satisfies

$$\begin{aligned}(\mathsf{row}(\mathtt{a}) \odot (\mathsf{row}(\mathtt{b}) \oplus \mathsf{row}(\mathtt{b})))(\square) &= (\mathsf{row}(\mathtt{a}) \odot \mathsf{row}(\mathtt{b} \parallel \mathtt{b}))(\square) \\ &= (\mathsf{row}(\mathtt{a}) \odot \mathsf{row}(1))(\square) = \mathsf{row}(\mathtt{a})(\square) = 0 \neq 1\end{aligned}$$

and thus recognises a language that differs from $\mathcal{L}$ on $\mathtt{a} \cdot (\mathtt{b} \parallel \mathtt{b}) \in T_{\langle S,E \rangle}$.

We thus have the following definition, parametric in a subset of $T_{\langle S,E \rangle}$.

**Definition 16 (Compatible hypothesis).** *A closed, consistent, and associative observation table $\langle S, E \rangle$ induces a hypothesis $\mathcal{H}$ that is $X$-*compatible *with its table, for $X \subseteq \mathsf{SP}$, if for $x \in X$ we have $x \in \mathcal{L}_\mathcal{H} \iff x \in \mathcal{L}$. We say that the hypothesis is* compatible *with its table if it is $T_{\langle S,E \rangle}$-compatible with its table.*

Ensuring hypotheses are compatible with their table will not be a crucial step in proving termination, but plays a key role in ensuring minimality (Section 3.4). This was originally shown by van Heerdt [14] for Mealy machines.

### 3.2 The Learning Algorithm

We are now ready to introduce our learning algorithm, Algorithm 1. The main algorithm initialises the table to $\langle \{1\}, \{\square\} \rangle$ and starts by augmenting the table to make sure it is closed and associative. We give an example below.

```
S = {1}, E = {□}
repeat
    repeat
        while ⟨S, E⟩ is not closed or not associative
            if ⟨S, E⟩ is not closed
                find t ∈ S⁺ such that row(t) ≠ row(s) for all s ∈ S
                S = S ∪ {t}
            for ♡ ∈ {·, ∥}
                if ⟨S, E⟩ is not ♡-associative
                    find s₁, s₂, s₃, s_l, s_r ∈ S and e ∈ E such that
                        row(s_l) = row(s₁ ♡ s₂),
                        row(s_r) = row(s₂ ♡ s₃), and
                        row(s_l ♡ s₃)(e) ≠ row(s₁ ♡ s_r)(e)
                    let b be the result of a membership query on s₁ ♡ s₂ ♡ s₃
                    if row(s_l ♡ s₃)(e) ≠ b
                        E = E ∪ {e[□ ♡ s₃]}
                    else
                        E = E ∪ {e[s₁ ♡ □]}
        construct the hypothesis ℋ for ⟨S, E⟩
        if ℋ is not compatible with its table
            find s ∈ S and e ∈ E such that e[s] ∈ ℒ_ℋ ⟺ e[s] ∉ ℒ
            E = E ∪ {HandleCounterexample(S, E, e[s], □)}
    until ℋ is compatible with its table
    if the teacher replies no to ℋ, with a counterexample z
        E = E ∪ {HandleCounterexample(S, E, z, □)}
until the teacher replies yes
return ℋ
```

```
HandleCounterexample(S, E, z, c)
if z ∈ S ∪ S⁺
    let s ∈ S be such that row(s) = row(z)
    if c[s] ∈ ℒ ⟺ c[z] ∈ ℒ
        return s
    else
        return c
let non-empty u₁, u₂ ∈ SP and ♡ ∈ {·, ∥} be such that u₁ ♡ u₂ = z
u₁ = HandleCounterexample(S, E, u₁, c[□ ♡ u₂])
if u₁ ∉ S
    return u₁
u₂ = HandleCounterexample(S, E, u₂, c[u₁ ♡ □])
if u₂ ∉ S
    return u₂
return HandleCounterexample(S, E, u₁ ♡ u₂, c)
```

Algorithm 1: The pomset recogniser learning algorithm.

*Example 17 (Fixing closedness and associativity).* Consider the table from Example 9, where $\mathsf{row}(\mathtt{aa}) \notin \{\mathsf{row}(1), \mathsf{row}(\mathtt{a}), \mathsf{row}(\mathtt{b})\}$ witnesses a closedness defect. To fix this, the algorithm would add $\mathtt{aa}$ to the set $S$, which means $\mathsf{row}(\mathtt{aa})$ will become part of the carrier of the hypothesis.

Now consider the table from Example 12. Here we found an associativity defect witnessed by $\mathsf{row}(\mathtt{a}) = \mathsf{row}(\mathtt{ab})$ and $\mathsf{row}(\mathtt{b}) = \mathsf{row}(\mathtt{ba})$ but $\mathsf{row}(\mathtt{aa}) \neq \mathsf{row}(\mathtt{ab})$. More specifically, $\mathsf{row}(\mathtt{aa})(\square) \neq \mathsf{row}(\mathtt{ab})(\square)$. Thus, $s_1 = s_3 = s_l = \mathtt{a}$, $s_2 = s_r = \mathtt{b}$, $s_l = \mathtt{a}$, and $e = \square$. A membership query on $\mathtt{aba}$ shows $\mathtt{aba} \notin \mathcal{L}$, so $b = 0$. We have $\mathsf{row}(\mathtt{aa})(\square) = 0$, and therefore the algorithm would add the context $\square[\mathtt{a} \cdot \square] = \mathtt{a} \cdot \square$ to $E$.

Note that the algorithm does not explicitly check for consistency; this is because we actually ensure a stronger property—sharpness [3]—as an invariant (Lemma 25). This property ensures every row indexed by a pomset in $S$ is indexed by exactly one pomset in $S$ (implying consistency):

**Definition 18 (Sharp table).** *An observation table $\langle S, E\rangle$ is* sharp *if for all $s_1, s_2 \in S$ such that $\mathsf{row}(s_1) = \mathsf{row}(s_2)$ we have $s_1 = s_2$.*

The idea of maintaining sharpness is due to Maler and Pnueli [29].

Once the table is closed and associative, we construct the hypothesis and check if it is compatible with its table. If this is not the case, a witness for incompatibility is a counterexample by definition, so HandleCounterexample is invoked to extract an extension of $E$, and we return to checking closedness and associativity. Once we obtain a hypothesis that is compatible with its table, we submit it to the teacher to check for equivalence with the target language. If the teacher provides a counterexample, we again process this and return to checking closedness and associativity. Once we have a compatible hypothesis for which there is no counterexample, we return this correct pomset recogniser.

The procedure HandleCounterexample, adapted from [7,8], is provided with an observation table $\langle S, E\rangle$ a pomset $z$, and a context $c$ and finds a single context to add to $E$. The main invariant is that $c[z]$ is a counterexample. Recursive calls replace subpomsets from $S^+$ with elements of $S$ in this counterexample while maintaining the invariant. There are two types of return values: if $c$ is a suitable context, $c$ is returned; otherwise the return value is an element of $S$ that is to replace $z$. The context $c$ is suitable if $z \in S^+$ and adding $c$ to $E$ would distinguish $\mathsf{row}(s)$ from $\mathsf{row}(z)$, where $s \in S$ is such that currently $\mathsf{row}(s) = \mathsf{row}(z)$. Because $S$ is non-empty and subpomset-closed, if $z \notin S \cup S^+$ it can be decomposed into $z = u_1 \heartsuit u_2$ for non-empty $u_1, u_2 \in \mathsf{SP}$ and $\heartsuit \in \{\cdot, \parallel\}$. We then recurse into $u_1$ and $u_2$ to replace them with elements of $S$ and replace $z$ with $u_1 \heartsuit u_2 \in S^+$ in a final recursive call. If $c = \square$, the return value cannot be in $S$, as we will show in Lemma 25 that these elements are not counterexamples.

*Example 19 (Processing a counterexample).* Consider $\mathcal{L} = \{\mathtt{a}, \mathtt{aa}, \mathtt{a} \parallel \mathtt{a}\}$, and let $S = \{1, \mathtt{a}\}$ and $E = \{\square\}$. This induces a closed, sharp, and associative table

| | $\square$ |
|---|---|
| 1 | 0 |
| $\mathtt{a}$ | 1 |

| | $\square$ |
|---|---|
| $\mathtt{aa}$ | 1 |
| $\mathtt{a} \parallel \mathtt{a}$ | 1 |

Suppose an equivalence query on its pomset recogniser, which rejects only the empty pomset, gives counterexample $z = \mathsf{a} \parallel \mathsf{a} \parallel \mathsf{aa}$. We may decompose $z$ as $(\square \parallel \mathsf{aa})[\mathsf{a} \parallel \mathsf{a}]$, where $\mathsf{a} \parallel \mathsf{a} \in S^+ \setminus S$. Because $\mathsf{row}(\mathsf{a} \parallel \mathsf{a}) = \mathsf{row}(\mathsf{a})$, $(\square \parallel \mathsf{aa})[\mathsf{a}] = \mathsf{a} \parallel \mathsf{aa}$, and $\mathsf{a} \parallel \mathsf{aa} \in \mathcal{L} \iff z \in \mathcal{L}$, we update $z = \mathsf{a} \parallel \mathsf{aa}$ and repeat the process. Now we decompose $z = (\mathsf{a} \parallel \square)[\mathsf{aa}]$. Since $\mathsf{row}(\mathsf{aa}) = \mathsf{row}(\mathsf{a})$, $(\mathsf{a} \parallel \square)[\mathsf{a}] = \mathsf{a} \parallel \mathsf{a}$, and $\mathsf{a} \parallel \mathsf{a} \in \mathcal{L} \iff z \notin \mathcal{L}$, we finish by adding $\mathsf{a} \parallel \square$ to $E$.

### 3.3 Termination and Query Complexity

Our termination argument is based on a comparison of the current observation table with the infinite table $\langle \mathsf{SP}, \mathsf{PC} \rangle$. We first show that the latter induces a hypothesis, called the *canonical pomset recogniser* for the language. Its underlying bimonoid is isomorphic to the syntactic bimonoid [28] for the language.

**Lemma 20.** *$\langle \mathsf{SP}, \mathsf{PC} \rangle$ is a closed, consistent, and associative observation table.*

**Definition 21 (Canonical pomset recogniser).** *The* canonical pomset recogniser *for $\mathcal{L}$ is the the hypothesis for the observation table $\langle \mathsf{SP}, \mathsf{PC} \rangle$. We denote this hypothesis by $\langle M_\mathcal{L}, \odot_\mathcal{L}, \oplus_\mathcal{L}, \mathbf{1}_\mathcal{L}, i_\mathcal{L}, F_\mathcal{L} \rangle$.*

The comparison of the current table with $\langle \mathsf{SP}, \mathsf{PC} \rangle$ is in terms of the number of distinct rows they hold. In the following lemma we show that the number of the former is bounded by the number of the latter.

**Lemma 22.** *If $M_\mathcal{L}$ is finite, any observation table $\langle S, E \rangle$ satisfies*

$$|\{\mathsf{row}(s) : s \in S\}| \leq |M_\mathcal{L}|.$$

*Proof.* Note that $M_\mathcal{L} = \{\mathsf{row}_{\langle \mathsf{SP},\mathsf{PC} \rangle}(s) : s \in S\}$. Given $s_1, s_2 \in S$ such that $\mathsf{row}_{\langle S,E \rangle}(s_1) \neq \mathsf{row}_{\langle S,E \rangle}(s_2)$ we have $\mathsf{row}_{\langle \mathsf{SP},\mathsf{PC} \rangle}(s_1) \neq \mathsf{row}_{\langle \mathsf{SP},\mathsf{PC} \rangle}(s_2)$. This implies $|\{\mathsf{row}(s) : s \in S\}| \leq |M_\mathcal{L}|$. □

An important fact will be that none of the pomsets in $S$ can form a counterexample for the hypothesis of a table $\langle S, E \rangle$. In order to show this we will first show that the hypothesis is always *reachable*, a concept we define for arbitrary pomset recognisers below.

**Definition 23 (Reachability).** *A pomset recogniser $\mathcal{R} = \langle M, \odot, \oplus, \mathbf{1}, i, F \rangle$ is* reachable *if for all $m \in M$ there exists $u \in \mathsf{SP}$ such that $i^\sharp(u) = m$.*

Our reachability lemma relies on the fact that $S$ is subpomset-closed.

**Lemma 24 (Hypothesis reachability).** *Given a closed, consistent, and associative observation table $\langle S, E \rangle$, the hypothesis it induces is reachable. In particular, ${i_H}^\sharp(s) = \mathsf{row}(s)$ for any $s \in S$.*

From the above it follows that we always have compatibility with respect to the set of row indices, as we show next.

**Lemma 25.** *The hypothesis of any closed, consistent, and associative observation table $\langle S, E\rangle$ is $S$-compatible.*

Before turning to our termination proof, we show that some simple properties hold throughout a run of the algorithm.

**Lemma 26 (Invariant).** *Throughout execution of Algorithm 1, we have that $\langle S, E\rangle$ is a sharp observation table.*

*Proof.* Subpomset-closedness holds throughout each run since $\{1\}$ is subpomset-closed and adding a single element of $S^+$ to $S$ preserves the property.

For sharpness, first note that the initial table is sharp as it only has one row. Sharpness of $\langle S, E\rangle$ can only be violated when adding elements to $S$. But the only place where this happens is on line 7, and there the new row is unequal to all previous rows, which means sharpness is preserved. □

The preceding results allow us to prove our termination theorem.

**Theorem 27 (Termination).** *If $M_{\mathcal{L}}$ is finite, then Algorithm 1 terminates.*

*Proof.* First, we observe that fixing a closedness defect by adding a row (line 7) can only happen finitely many times, since, by Lemma 22, the size of $\{\mathsf{row}(s) : s \in S\}$ is bounded by $M_{\mathcal{L}}$.

This means that it suffices to show the following two points:

1. Each iteration of any of the loops starting on lines 2–4 either fixes a closedness defect by adding a row, or adapts $E$ so that $\langle S, E\rangle$ ends up *not* being closed at the end of loop body. In the second case, a closedness defect will be fixed in the following iteration of the inner while loop.
2. The calls to HandleCounterexample terminate.

Combined, these show that the algorithm terminates. For the first point, we treat each of the cases:

- If the table is not closed, we directly find a new row that is taken from the $S^+$-part of the table and added to the $S$-part of the table.
- Consider the failure of $\heartsuit$-associativity, for $\heartsuit \in \{\cdot, \parallel\}$, and let $s_1, s_2, s_3, s_l, s_r \in S$ and $e \in E$ be such that $\mathsf{row}(s_l) = \mathsf{row}(s_1 \heartsuit s_2)$, $\mathsf{row}(s_r) = \mathsf{row}(s_2 \heartsuit s_3)$, and $\mathsf{row}(s_l \heartsuit s_3)(e) \neq \mathsf{row}(s_1 \heartsuit s_r)(e)$. Suppose $\mathsf{row}(s_l \heartsuit s_3)(e) \neq b$, with $b$ be the result of a membership query on $s_1 \heartsuit s_2 \heartsuit s_3$. Then $e[\square \heartsuit s_3]$ distinguishes the previously equal rows $\mathsf{row}(s_1 \heartsuit s_2)$ and $\mathsf{row}(s_l)$, so adding it to $E$ creates a closedness defect. The fact that $\mathsf{row}(s_1 \heartsuit s_2)$ cannot remain equal to another row than $\mathsf{row}(s_l)$ is a result of the sharpness invariant.
  Alternatively, $\mathsf{row}(s_l \heartsuit s_3)(e) = b$ means $\mathsf{row}(s_1 \heartsuit s_r)(e) \neq b$, for otherwise we would contradict $\mathsf{row}(s_l \heartsuit s_3)(e) \neq \mathsf{row}(s_1 \heartsuit s_r)(e)$. For similar reasons the context $e[s_1 \heartsuit \square]$ in this case distinguishes the previously equal rows $\mathsf{row}(s_1 \heartsuit s_2)$ and $\mathsf{row}(s_r)$, creating a closedness defect.
- A compatibility defect results in the identification of a counterexample, the handling of which we discuss next.

– Whenever a counterexample is identified, we eventually find a context $c$, $s \in S$, and $t \in S^+ \setminus S$ such that $\mathsf{row}(t) = \mathsf{row}(s)$ and $c[t] \in \mathcal{L} \iff c[s] \notin \mathcal{L}$. Thus, adding $c$ to $E$ creates a closedness defect.

Termination of HandleCounterexample follows: the first two recursive calls in the procedure replace $z$ with strict subpomsets of $z$, whereas the last one replaces $z$ with an element of $S^+$, so no further recursion will happen. □

*Query Complexity.* We determine upper bounds on the membership and equivalence query numbers of a run of the algorithm in terms of the size of the canonical pomset recogniser $n = |M_{\mathcal{L}}|$, the size of the alphabet $k = |\Sigma|$, and the maximum number of operations (from $\{\cdot, \|\}$, used to compose alphabet symbols) $m$ found in a counterexample. We note that since the number of distinct rows indexed by $S$ is bounded by $n$ and the table remains sharp throughout any run, the final size of $S$ is at most $n$. Thus, the final size of $S^+$ is in $\mathcal{O}(n^2 + k)$. Given the initialisation of $S$ with a single element, the number of closedness defects fixed throughout a run is at most $n - 1$. This means that the total number of associativity defects fixed and counterexamples handled (including those resulting from compatibility defects) together is $n - 1$. We can already conclude that the number of equivalence queries posed is bounded by $n$. Moreover, we know that the final table will have at most $n$ columns, and therefore the total number of cells in that table will be in $\mathcal{O}(n^3 + kn)$.

The number of membership queries posed during a run of the algorithm is given by the number of cells in the table plus the number of queries needed during the processing of counterexamples. Consider the counterexample $z$ that contains the maximum number of operations among those encountered during a run. The first two recursive calls of HandleCounterexample break down one operation, whereas the third is used to execute a base case making two membership queries and does not lead to any further recursion. The number of membership queries made starting from a given counterexample is thus in $\mathcal{O}(m)$. This means the total number of membership queries during the processing of counterexamples is in $\mathcal{O}(mn)$, from which we conclude that the number of membership queries posed during a run is in $\mathcal{O}(n^3 + mn + kn)$.

### 3.4 Minimality of Hypotheses

In this section we will show that all hypotheses submitted by the algorithm to the teacher are minimal. We first need to define what minimality means. As is the case for DFAs, it is the combination of an absence of unreachable states and of every state exhibiting its own distinct behaviour.

**Definition 28 (Minimality).** *A pomset recogniser $\mathcal{R} = \langle M, \odot, \oplus, \mathbf{1}, i, F\rangle$ is* minimal *if it is reachable and for all $u, v \in \mathsf{SP}$ with $i^\sharp(u) \neq i^\sharp(v)$ there exists $c \in \mathsf{PC}$ such that $c[u] \in \mathcal{L}_{\mathcal{R}} \iff c[v] \notin \mathcal{L}_{\mathcal{R}}$.*

Before proving the main result of this section, we need the following:

**Lemma 29.** *For all pomset recognisers* $\langle M, \odot, \oplus, \mathbf{1}, i, F\rangle$ *and* $u, v \in \mathsf{SP}$ *such that* $i^\sharp(u) = i^\sharp(v)$ *we have for any* $c \in \mathsf{PC}$ *that* $i^\sharp(c[u]) = i^\sharp(c[v])$*.*

The minimality theorem below relies on table compatibility, which allows us to distinguish the behaviour of states based on the contents of their rows. Note that the algorithm only submits a hypothesis in an equivalence query if that hypothesis is compatible with its table.

**Theorem 30 (Minimality of hypotheses).** *A closed, consistent, and associative observation* $\langle S, E\rangle$ *induces a minimal hypothesis if the hypothesis is compatible with its table.*

*Proof.* We obtain the hypothesis from Lemma 14. Since $S$ is subpomset-closed, we have by Lemma 24 that the hypothesis is reachable. Moreover, for every $s \in S$ we have ${i_H}^\sharp(s) = \mathsf{row}(s)$. Consider $u_1, u_2 \in \mathsf{SP}$ such that ${i_H}^\sharp(u_1) \neq {i_H}^\sharp(u_2)$. Then there exist $s_1, s_2 \in S$ such that $\mathsf{row}(s_1) = {i_H}^\sharp(u_1)$ and $\mathsf{row}(s_2) = {i_H}^\sharp(u_2)$, and we have $\mathsf{row}(s_1) \neq \mathsf{row}(s_2)$. Let $e \in E$ be such that $\mathsf{row}(s_1)(e) \neq \mathsf{row}(s_2)(e)$. We have

$$\begin{aligned}
{i_H}^\sharp(e[u_1]) \in F_H &\iff {i_H}^\sharp(e[s_1]) \in F_H && \text{(Lemma 29)}\\
&\iff e[s_1] \in \mathcal{L}_\mathcal{H}\\
&\iff \mathsf{row}(s_1)(e) = 1\\
&\iff \mathsf{row}(s_2)(e) = 0\\
&\iff e[s_2] \notin \mathcal{L}_\mathcal{H}\\
&\iff {i_H}^\sharp(e[s_2]) \notin F_H\\
&\iff {i_H}^\sharp(e[u_2]) \notin F_H. && \text{(Lemma 29)} \quad \square
\end{aligned}$$

As a corollary, we find that the canonical pomset recogniser is minimal.

**Proposition 31.** *The canonical pomset recogniser is minimal.*

## 4 Conversion to Pomset Automata

Bimonoids are a useful representation of pomset languages because sequential and parallel composition are on an equal footing; in the case of the learning algorithm of the previous section, this helps us treat both operations similarly. On the other hand, the behaviour of a program is usually thought of as a series of actions, some of which involve launching two or more threads that later combine. Here, sequential actions form the basic unit of computation, while fork/join patterns of threads are specified separately. *Pomset automata* [22] encode this more asymmetric model: they can be thought of as non-deterministic finite automata with an additional transition type that brokers forking and joining threads.

In this section, we show how to convert a pomset recogniser to a certain type of pomset automaton, where acceptance of a pomset is guided by its structure; conversely, we show that each of the pomset automata in this class can be represented by a pomset recogniser. Together with the previous section, this establishes that the languages of pomset automata in this class are learnable.

If $S$ is a set, we write $\mathbb{M}(S)$ for the set of *finite multisets* over $S$. A finite multiset over $S$ is written $\phi = \{\!|s_1, \ldots, s_n|\!\}$.

**Definition 32 (Pomset automata).** *A* pomset automaton *(PA) is a tuple* $A = \langle Q, I, F, \delta, \gamma \rangle$ *where*

- $Q$ *is a set of* states*, with* $I, F \subseteq Q$ *the* initial *and* accepting states*, and*
- $\delta\colon Q \times \Sigma \to 2^Q$ *the* sequential transition function*, and*
- $\gamma\colon Q \times \mathbb{M}(Q) \to 2^Q$ *the* parallel transition function.

*Lastly, for every* $q \in Q$ *there are finitely many* $\phi \in \mathbb{M}(Q)$ *such that* $\gamma(q, \phi) \neq \emptyset$.

A finite PA can be represented graphically: every state is drawn as a vertex, with accepting states doubly circled and initial states pointed out by an arrow, while $\delta$-transitions are represented by labelled edges, and $\gamma$-transitions are drawn as a multi-ended edge. For instance, in Figure 1a, we have drawn a PA with states $q_0$ through $q_5$ with $q_5$ accepting, and $q_1 \in \delta(q_0, \mathtt{a})$ (among other $\delta$-transitions), while the multi-ended edge represents that $q_2 \in \gamma(q_1, \{\!|q_3, q_4|\!\})$, i.e., $q_2$ can launch threads starting in $q_3$ and $q_4$, which, upon termination, resume in $q_2$.

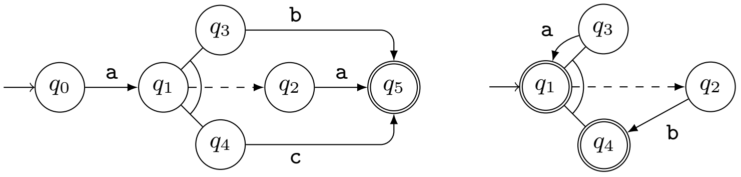

(a) A simple PA. (b) A non-saturated PA.

Fig. 1: Some pomset automata.

The sequential transition function is interpreted as in non-deterministic finite automata: if $q' \in \delta(q, \mathtt{a})$, then a machine in state $q$ may transition to state $q'$ after performing the action $\mathtt{a}$. The intuition to the parallel transition function is that if $q' \in \gamma(q, \{\!|r_1, \ldots, r_n|\!\})$, then a machine in state $q$ may launch threads starting in states $r_1$ through $r_n$, and when each of those has terminated succesfully, may proceed in state $q'$. Note how the representation of starting states in a $\gamma$-transition allows for the possibility of launching multiple instances of the same thread, and disregards their order—i.e., $\gamma(q, \{\!|r_1, \ldots, r_n|\!\}) = \gamma(q, \{\!|r_n, \ldots, r_1|\!\})$. This intuition is made precise through the notion of a *run*.

**Definition 33 (Run relation).** *The* run relation *of a PA* $A = \langle Q, I, F, \delta, \gamma \rangle$*, denoted* $\to_A$*, is defined as the the smallest subset of* $Q \times \mathsf{SP} \times Q$ *satisfying*

$$\frac{}{q \xrightarrow{1}_A q} \qquad \frac{q' \in \delta(q, \mathtt{a})}{q \xrightarrow{\mathtt{a}}_A q'} \qquad \frac{\forall 1 \leq i \leq n.\ r_i \xrightarrow{u_i}_A r_i' \in F \quad q' \in \gamma(q, \{\!|r_1, \ldots, r_n|\!\})}{q \xrightarrow{u_1 \parallel \cdots \parallel u_n}_A q'} \qquad \frac{q \xrightarrow{u}_A q'' \quad q'' \xrightarrow{v}_A q'}{q \xrightarrow{u \cdot v}_A q'}$$

*The* language accepted *by* $A$ *is* $\mathcal{L}_A = \{u \in \mathsf{SP} : \exists q \in I, q' \in F.\ q \xrightarrow{u}_A q'\}$.

*Example 34.* If $A$ is the PA from Figure 1a, we can see that $q_3 \xrightarrow{\mathtt{b}}_A q_5$ and $q_4 \xrightarrow{\mathtt{c}}_A q_5$ as a result of the second rule; by the third rule, we find that $q_1 \xrightarrow{\mathtt{b} \parallel \mathtt{c}}_A q_2$. Since $q_2 \xrightarrow{\mathtt{a}} q_5$ and $q_0 \xrightarrow{a}_A q_1$ (again by the second rule), we can conclude $q_0 \xrightarrow{\mathtt{a} \cdot (\mathtt{b} \parallel \mathtt{c}) \cdot \mathtt{a}}_A q_5$ by repeated application of the last rule. The language accepted by this PA is the singleton set $\{\mathtt{a} \cdot (\mathtt{b} \parallel \mathtt{c}) \cdot \mathtt{a}\}$.

In general, finite pomset automata can accept a very wide range of pomset languages, including all context free (pomset) languages [23]. The intuition behind this is that the mechanism of forking and joining encoded in $\gamma$ can be used to simulate a call stack. For example, the automaton in Figure 1b accepts the strictly context-free language (of words) $\{\mathtt{a}^n \cdot \mathtt{b}^n : n \in \mathbb{N}\}$. It follows that PAs can represent strictly more pomset languages than pomset recognisers. To tame the expressive power of PAs at least slightly, we propose the following.

**Definition 35 (Saturation).** *We say that* $A = \langle Q, I, F, \delta, \gamma\rangle$ *is* saturated *when for all* $u, v \in \mathsf{SP}$ *with* $u, v \neq 1$*, both of the following are true:*

*(i) If* $q \xrightarrow{u \cdot v}_A q'$*, then there exists a* $q'' \in Q$ *with* $q \xrightarrow{u}_A q''$ *and* $q'' \xrightarrow{v}_A q'$.
*(ii) If* $q \xrightarrow{u \parallel v}_A q'$*, then there exist* $r, s \in Q$ *and* $r', s' \in F$ *such that*

$$r \xrightarrow{u}_A r' \qquad\qquad s \xrightarrow{v}_A s' \qquad\qquad q' \in \gamma(q, \{\!|r, s|\!\})$$

*Example 36.* Returning to Figure 1, we see that the PA in Figure 1a is saturated, while Figure 1b is not, as a result of the run $q_1 \xrightarrow{\mathtt{a}\cdot\mathtt{a}\cdot\mathtt{b}\cdot\mathtt{b}}_A q_4$, which does not admit an intermediate state $q$ such that $q_1 \xrightarrow{\mathtt{a}\cdot\mathtt{a}}_A q$ and $q \xrightarrow{\mathtt{b}\cdot\mathtt{b}}_A q_4$.

We now have everything in place to convert the encoding of a language given by a pomset recogniser to a pomset automaton. The idea is to represent every element $q$ of the bimonoid by a state which accepts exactly the language of pomsets mapped to $q$; the transition structure is derived from the operations.

**Lemma 37.** *Let* $\mathcal{R} = \langle M, \odot, \oplus, \mathbf{1}, i, F\rangle$ *be a pomset recogniser. We construct the pomset automaton* $A = \langle M, F, \{\mathbf{1}\}, \delta, \gamma\rangle$ *(note: we use* $F$ *as the set of initial states) where* $\delta \colon M \times \Sigma \to 2^M$ *and* $\gamma \colon M \times \mathbb{M}(M) \to 2^M$ *are given by*

$$\delta(q, \mathtt{a}) = \{q' : i(\mathtt{a}) \odot q' = q\} \qquad \gamma(q, \phi) = \{q' : (r \oplus r') \odot q' = q,\ \phi = \{\!|r, r'|\!\}\}$$

*Then* $A$ *is saturated, and* $\mathcal{L}_A = \mathcal{L}_{\mathcal{R}}$.

*Example 38.* Let $\langle M, \odot, \oplus, \mathbf{1}, i, F\rangle$ be the pomset recogniser from Example 7. The pomset automaton that arises from the construction above is partially depicted in Figure 2; we have not drawn the state $q_\perp$ and its incoming transitions, or forks into $\mathbf{1}$, to avoid clutter. In this PA, we see that, since $q_\mathtt{a} \odot q_1 = q_\mathtt{b}$ and $i(\mathtt{a}) = q_\mathtt{a}$, we have $q_1 \in \delta(q_\mathtt{b}, \mathtt{a})$. Furthermore, since $(q_\mathtt{b} \oplus q_\mathtt{b}) \odot \mathbf{1} = q_1 \odot \mathbf{1} = q_1$, we also have $\mathbf{1} \in \gamma(q_1, \{\!|q_\mathtt{b}, q_\mathtt{b}|\!\})$. Finally, $q_\mathtt{b}$ is initial, since $F = \{q_\mathtt{b}\}$.

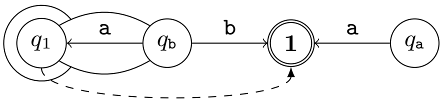

Fig. 2: Part of the PA obtained from the pomset recogniser from Example 7, using the construction from Lemma 37. The state $q_\perp$ (which does not contribute to the language of the automaton) and forks into the state **1** are not pictured.

We have thus shown that the language of any pomset recogniser can be accepted by a finite and saturated PA. In turn, this shows that our algorithm can, in principle, be adapted to work with a teacher that takes a (saturated) PA instead of a pomset recogniser as hypothesis, by simply converting the hypothesis pomset recogniser to an equivalent PA before sending it over.

Conversely, we can show that the transition relations of a saturated PA carry the algebraic structure of a bimonoid, and use that to show that a language recognised by a saturated PA is also recognised by a bimonoid. This shows that our characterisation is "tight", i.e., languages recognised by saturated PAs are precisely those recognised by bimonoids, and hence learnable.

**Lemma 39.** *Let $A = \langle Q, I, F, \delta, \gamma \rangle$ be a saturated pomset automaton. We can construct a pomset recogniser $\mathcal{R} = \langle M, \odot, \oplus, \mathbf{1}, i, F' \rangle$, where*

$$M = \{ \xrightarrow{u}_A : u \in \mathsf{SP} \} \qquad \xrightarrow{u}_A \odot \xrightarrow{v}_A = \xrightarrow{u \cdot v}_A \qquad \xrightarrow{u}_A \oplus \xrightarrow{v}_A = \xrightarrow{u \parallel v}_A$$

$$i(\mathtt{a}) = \xrightarrow{\mathtt{a}}_A \qquad F' = \{ \xrightarrow{u}_A \in M : \exists q \in I, q' \in F.\ q \xrightarrow{u}_A q' \}$$

*Now $\odot$ and $\oplus$ are well-defined, and $\mathcal{R}$ is a pomset recogniser such that $\mathcal{L}_\mathcal{R} = \mathcal{L}_A$.*

If $A$ is finite, then so is $\mathcal{R}$, since each of the elements of $M$ is a relation on $Q$, and there are finitely many relations on a finite set.

In general, the PA obtained from a pomset recogniser may admit runs where the same fork transition is nested repeatedly. Recognisable pomset languages of *bounded width* may be recognised by a pomset recogniser that is *depth-nilpotent* [28], which can be converted into a *fork-acyclic* PA by way of an sr-expression [28,22]. However, this detour via sr-expressions is not necessary: one can adapt Lemma 37 to produce a fork-acyclic PA, when given a depth-nilpotent pomset recogniser. The details are discussed in the full version [15].

We conclude this section by remarking that the minimal pomset recogniser for a bounded-width language is necessarily depth-nilpotent [28]; since our algorithm produces a minimal pomset recogniser, this means that we can also produce a fork-acyclic PA after learning a bounded-width recognisable pomset language.

## 5 Discussion

To learn DFAs, there are several alternatives to the observation table data structure that reduce the space complexity of the algorithm. Most notable is the *classification tree* [25], which distinguishes individual pairs of words (which for us would be pomsets) at every node rather than filling an entire row for each of them. The TTT algorithm [19] further builds on this and achieves optimal space complexity. Given that we developed the first learning algorithm for pomset languages, we opted for the simplicity of the observation table—optimisations such as those analogous to the aforementioned work are left to future research.

We would like to extend our algorithm to learn recognisers based on arbitrary algebraic theories. One challenge is to ensure that the equations of the theory hold for hypotheses, by generalising our definition of associativity (Definition 13).

Our algorithm can also be specialised to learn languages recognised by commutative monoids. These languages of *multisets* can alternatively be represented as semi-linear sets [30] or described using Presburger arithmetic [11]. While not all languages described this way are recognisable (for instance, the set of multisets over $\Sigma = \{\mathtt{a}, \mathtt{b}\}$ with as many $\mathtt{a}$'s as $\mathtt{b}$'s [28]), it would be interesting to be able to learn at least the fragment representable by commutative monoids, and apply that to one of the domains where semi-linear sets are used.

Our algorithm is limited to learning languages of series-parallel pomsets; there exist pomsets which are not series-parallel, each of which must contain an "N-shape" [12,13,35]. Since N-shapes appear in pomsets that describe message passing between threads, we would like to be able to learn such languages as well. We do not see an obvious way to extend our algorithm to include these pomsets, but perhaps recent techniques from [10] can provide a solution.

Every hypothesis of our algorithm can be converted to a pomset automaton. The final pomset recogniser for a bounded-width language is minimal, and hence depth-nilpotent [28], which means that it can be converted to a fork-acyclic PA. In future work, we would like to guarantee that the same holds for intermediate hypotheses when learning a bounded-width language.

Running two threads in parallel may be implemented by running some initial section of those threads in parallel, followed by running the remainder of those threads in parallel. This interleaving is represented by the *exchange law* [12,13]. One can specialise pomset recognisers to include this interleaving to obtain recognisers of pomset languages closed under subsumption [28], i.e., such that if a pomset $u$ is recognised, then so are all of the "more sequential" versions of $u$. We would like to adapt our algorithm to learn these types of recognisers, and exploit the extra structure provided by the exchange law to optimise further.

We have shown that recognisable pomset languages correspond to saturated regular pomset languages (Lemmas 37 and 39). One question that remains is whether there is an algorithm that can learn all or at least a larger class of regular pomset languages. Given that pomset automata can accept context-free languages (Figure 1b), we wonder if a suitable notion of context-free grammars for pomset languages could be identified. Clark [6] showed that there exists a subclass of context-free languages that can be learned via an adaptation of $\mathtt{L}^\star$. Arguably, this adaptation learns recognisers with a monoidal structure and reverses this structure to obtain a grammar. An extension of this work to pomset languages might lead to a learning algorithm that learns more PAs.
